# Preserve a Voucher Specimen! The Critical Need for Integrating Natural History Collections in Infectious Disease Studies

**DOI:** 10.1128/mBio.02698-20

**Published:** 2021-01-12

**Authors:** Cody W. Thompson, Kendra L. Phelps, Marc W. Allard, Joseph A. Cook, Jonathan L. Dunnum, Adam W. Ferguson, Magnus Gelang, Faisal Ali Anwarali Khan, Deborah L. Paul, DeeAnn M. Reeder, Nancy B. Simmons, Maarten P. M. Vanhove, Paul W. Webala, Marcelo Weksler, C. William Kilpatrick

**Affiliations:** a Department of Ecology and Evolutionary Biology, University of Michigan, Ann Arbor, Michigan, USA; b Museum of Zoology, University of Michigan, Ann Arbor, Michigan, USA; c EcoHealth Alliance, New York, New York, USA; d Center of Food Safety and Applied Nutrition, U. S. Food and Drug Administration, College Park, Maryland, USA; e Museum of Southwestern Biology, Biology Department, University of New Mexico, Albuquerque, New Mexico, USA; f Gantz Family Collections Center, Field Museum of Natural History, Chicago, Illinois, USA; g Gothenburg Natural History Museum, Gothenburg, Sweden; h Gothenburg Global Biodiversity Centre, Gothenburg, Sweden; i Faculty of Resource Science and Technology, Universiti Malaysia Sarawak, Sarawak, Malaysia; j Florida State University, Tallahassee, Florida, USA; k Species File Group, University of Illinois, Urbana-Champaign, Illinois, USA; l Bucknell University, Lewisburg, Pennsylvania, USA; m Department of Mammalogy, Division of Vertebrate Zoology, American Museum of Natural History, New York, New York, USA; n Hasselt University, Centre for Environmental Sciences, Research Group Zoology: Biodiversity and Toxicology, Diepenbeek, Belgium; o Department of Forestry and Wildlife Management, Maasai Mara University, Narok, Kenya; p Departamento de Vertebrados, Museu Nacional, Universidade Federal do Rio de Janeiro, Rio de Janeiro, Brazil; q University of Vermont, Burlington, Vermont, USA; Albert Einstein College of Medicine

**Keywords:** biorepositories, coronaviruses, extended specimen, holistic specimen, museums, zoonoses

## Abstract

Despite being nearly 10 months into the COVID-19 (coronavirus disease 2019) pandemic, the definitive animal host for SARS-CoV-2 (severe acute respiratory syndrome coronavirus 2), the causal agent of COVID-19, remains unknown. Unfortunately, similar problems exist for other betacoronaviruses, and no vouchered specimens exist to corroborate host species identification for most of these pathogens. This most basic information is critical to the full understanding and mitigation of emerging zoonotic diseases. To overcome this hurdle, we recommend that host-pathogen researchers adopt vouchering practices and collaborate with natural history collections to permanently archive microbiological samples and host specimens. Vouchered specimens and associated samples provide both repeatability and extension to host-pathogen studies, and using them mobilizes a large workforce (i.e., biodiversity scientists) to assist in pandemic preparedness. We review several well-known examples that successfully integrate host-pathogen research with natural history collections (e.g., yellow fever, hantaviruses, helminths). However, vouchering remains an underutilized practice in such studies. Using an online survey, we assessed vouchering practices used by microbiologists (e.g., bacteriologists, parasitologists, virologists) in host-pathogen research. A much greater number of respondents permanently archive microbiological samples than archive host specimens, and less than half of respondents voucher host specimens from which microbiological samples were lethally collected. To foster collaborations between microbiologists and natural history collections, we provide recommendations for integrating vouchering techniques and archiving of microbiological samples into host-pathogen studies. This integrative approach exemplifies the premise underlying One Health initiatives, providing critical infrastructure for addressing related issues ranging from public health to global climate change and the biodiversity crisis.

## Abstract (precis)

Despite being nearly 10 months into the COVID-19 (coronavirus disease 2019) pandemic, the definitive animal host for SARS-CoV-2 (severe acute respiratory syndrome coronavirus 2), the causal agent of COVID-19, remains unknown. Unfortunately, similar problems exist for other betacoronaviruses, and no vouchered specimens exist to corroborate host species identification for most of these pathogens.

## INTRODUCTION

The current world focus on COVID-19 (coronavirus disease 2019) has brought considerable interest and urgency to efforts to better understand emerging infectious diseases (EIDs), particularly those that threaten public health. A significant increase in the transmission and emergence of infectious diseases attributed to novel pathogens that spill over from animal populations, including domesticated and wild taxa, has been noted in recent decades (1, 2). SARS-CoV-2 (severe acute respiratory syndrome coronavirus 2) (3), the causative agent of COVID-19, has demonstrated that a previously unknown pathogen can emerge from wildlife species and threaten public health on a global scale within months. SARS-CoV-2 belongs to a clade of betacoronaviruses that have been detected in rhinolophid bats (4–6) and pangolins (4, 7, 8). SARS-CoV-2 was initially recognized by Wu et al. (9) as similar to viruses previously reported from intestinal tissue of Chinese horseshoe bats, Rhinolophus sinicus, despite mitochondrial cytochrome *b* sequences identifying the bats sampled as least horseshoe bats (Rhinolophus pusillus) elsewhere in the same publication (10). However, the exact animal source of the spillover of SARS-CoV-2 into human populations remains unknown (4, 11).

Despite numerous studies of SARS-CoV-2, the diversity of coronaviruses in bats and other wildlife species remains understudied. Only 19 coronaviruses had been described prior to the SARS epidemic in 2002 (12). Multiple research groups subsequently surveyed wildlife populations for SARS-CoV, the virus that causes the human disease SARS (13–27). Unfortunately, none of these studies included the deposition of vouchered host specimens for SARS-CoV (or any other coronaviruses detected during the surveys) in natural history collections, creating a large gap in our ability to further probe the diversity, distribution, and basic biology responsible for the transmission of SARS and SARS-like viruses in wildlife and human populations and the evolutionary processes by which viruses with pandemic potential evolve in their reservoir hosts. Lack of basic biodiversity infrastructure, in the form of vouchered specimen collections, has limited our ability to respond to the current COVID-19 pandemic yet is critically needed to better understand other zoonotic pathogens with pandemic potential (28).

Host vouchering (i.e., collection and archiving of host species and/or their tissues in a permanent repository) in most recent zoonotic pathogen studies has effectively been nonexistent, including studies relevant to understanding the origins of SARS-CoV-2. For example, Hu et al. (10) euthanized and necropsied over 300 horseshoe bats (genus *Rhinolophus*) while screening for coronaviruses, yet no host voucher specimen(s) or archived tissue samples were reportedly preserved. None of the cytochrome *b* sequences from R. pusillus (*n* = 47) or R. sinicus (*n* = 206) in GenBank as of June 2020 can be linked to the bats examined by Hu et al. (10). To our knowledge, none of the 30 studies describing sequences of the subgenus *Sarbecovirus* (genus *Betacoronavirus*)—which includes SARS-CoV-2—from wild mammalian hosts provided any information concerning permanently vouchered material (i.e., 5, 7, 10, 14–27, 29–41).

The same pattern applies to studies of betacoronaviruses. Few studies describing the recognized viral lineages isolated from wild mammal reservoirs have associated host specimens archived in a natural history collection (but see references 42 and 43). Most published studies of betacoronaviruses used oropharyngeal and rectal swabs collected directly from wild mammals or sampled feces or urine samples from caves or other bat dwellings (34, 44–52). Voucher specimens were either not collected or archived.

We are aware of only a single study that explicitly mentions the use of vouchered host specimens as part of an analysis of coronavirus diversity in bats—a study conducted in the western Indian Ocean (43). The vouchered hosts and corresponding tissue samples were initially deposited in natural history collections as part of previous host-pathogen research (53–56). As shown by Joffrin et al. (43), natural history collections, including associated collections of cryopreserved microbiological samples and other tissues, have the potential to promote powerful interdisciplinary and historical approaches to studying emerging zoonotic pathogens (28). Archived specimens represent a vast, largely untapped, biodiversity infrastructure that can facilitate pathogen identification and insights into host ecology, immunology, and host-pathogen coevolution and dynamics, which may be necessary ingredients for disease prediction (28). In this context, we (i) review the importance of host voucher specimens and microbiological samples in host-pathogen research, (ii) examine vouchering practices in the host-pathogen research community, (iii) provide examples of successful integration of natural history collections in studies of infectious diseases, and (iv) outline recommendations for engaging natural history collections as resources in preventing future pandemics.

## IMPORTANCE OF VOUCHERING IN HOST-PATHOGEN STUDIES

### Making the most of every sampling event.

Microbiologists and other researchers that conduct pathobiology studies typically collect target samples—bacteria, protozoans, viruses, or fungal pathogens—by capturing and sampling wildlife species. While many microbiological samples do not require sacrificing the host to collect samples (e.g., blood, urine, feces, oral and rectal swabs), others may require lethal sampling (e.g., heart, liver, spleen, brain, and digestive tract) (57). Even when intentional lethal sampling is not a goal of a study, there are frequently accidental deaths of animals during capture and handling (58, 59), and it is poor scientific practice and unethical if resulting carcasses and/or additional microbiological samples are not permanently archived so that additional insights can come from the deceased animal (60). Regardless of the goals, studies involving wildlife species should preserve and archive additional sample types from the host, in addition to the target microbiological sample for pathogen detection, to properly document the symbiotic relationship and identity of the host. This is true even when terminal sampling is not required to achieve the study goals (e.g., when only swabs or blood samples are taken from the host). If only a few voucher specimens or tissue samples (e.g., “representative samples”) are collected and archived during a particular study, these samples provide a means of ensuring correct host identification now and in the future, especially important in species-rich taxa (e.g., bats, rodents), where taxonomy is uncertain and archived materials would provide a resource for future studies beyond the focus of the initial study.

### The voucher specimen—a tool for pandemic preparedness.

Voucher specimens have long been the backbone of biodiversity science (61–63). Over the last few decades, natural history collections have embraced the holistic or extended specimen concept (64, 65) whereby associated parasites and diverse cryogenically preserved tissues are associated with each host specimen. The utility of voucher specimens has expanded in novel ways from their traditional use in systematics and taxonomy to studies that now also link together stable isotope analyses, next-generation sequencing, and X-ray computed tomography (CT) scanning (66). Despite these novel uses, the hallmark features of voucher specimens have remained unchanged—to serve as a physical record for biodiversity (67), allowing for verification and documentation of species identifications and distributions, ensuring repeatability in scientific studies, and allowing subsequent researchers to reexamine and reevaluate archived material long after their initial collection (68).

Vouchering in host-pathogen research is necessary for the same reasons that it is important in studies of macroscopic organisms; indeed, it may be more important since hosts effectively represent the habitat of pathogens. Identification of both the pathogen and host is critical to fully understand the biology, ecology, evolution, and potential for transmission of pathogens to other species, including humans and domesticated animals and plants (69). Without depositing host voucher specimens and/or tissue samples in natural history collections or other biorepositories, a host-pathogen study becomes difficult or impossible to repeat and hence is potentially not verifiable by other researchers (68, 70). Furthermore, any possibility of isolating additional coinfecting pathogens is lost when voucher specimens are not collected and properly archived. This problem becomes especially critical in navigating novel viral zoonoses, such as the COVID-19 pandemic, where it is necessary for the scientific community to swiftly and efficiently leverage its collective knowledge and resources to effectively understand and contain the spread of novel pathogens at a time when lockdown restrictions hamper on-going collecting efforts (28).

Despite the clear utility of vouchering in host-pathogen research, especially research focused on EIDs, there are other significant benefits to vouchering that accrue from the process of archiving samples in natural history collections or biorepositories. Vouchered host specimens provide scientific replicability, baselines for temporal and spatial studies, correct taxonomic identification of host species (which may be later revised, e.g., see reference 71), and material for extending research using advanced technologies. Vouchering also integrates an entire community of researchers into the public health sector that otherwise would not typically collaborate (72) and provides critical expertise about the ecology and evolution of host species to support the One Health model for tackling EIDs (73). This proactive approach allows the sharing of data and expertise as well as fostering collaborations to collectively develop novel, creative insights that provide substantial benefits across diverse scientific fields. In essence, vouchering provides both an offensive mechanism to pandemic prevention by expanding the surveillance of wildlife hosts and associated pathogens, in addition to a defensive mechanism by providing a verifiable temporal and spatial archive for baseline comparisons.

## INSIGHTS INTO CURRENT VOUCHERING PRACTICES IN HOST-PATHOGEN RESEARCH

We explored current vouchering practices commonly employed by microbiologists (e.g., virologists, bacteriologists, parasitologists) in host-pathogen research and existence of collaborations with natural history collections based on an online survey (see Text S1 in the supplemental material for survey methodology, institutional review board [IRB] approval, and summarized data). Herein we discuss responses from 109 completed surveys (see Data Set S1 in the supplemental material for raw survey results). Distribution of the survey through social media resulted in a broad range of respondents from all continents except Antarctica. Participants were asked to classify their field(s) of research, and 38.5% indicated two or more disciplines, reflecting the multidisciplinary nature of microbiological research today. Not surprising given the diversity of respondents, 39.4% indicated studying more than one taxonomic group. Helminths (e.g., monogeneans, tapeworms, nematodes, leeches) were the most frequently cited focal taxa, followed by bacteria.

10.1128/mBio.02698-20.1 TEXT S1 Microbiology sampling methods and museums—a human survey. Download Text S1, DOCX file, 0.03 MB. Copyright © 2021 Thompson et al. 2021 Thompson et al. This content is distributed under the terms of the Creative Commons Attribution 4.0 International license.

10.1128/mBio.02698-20.2 DATA SET S1 Microbiology sampling methods and museums—a human survey. Download Data Set S1, XLSX file, 0.03 MB. Copyright © 2021 Thompson et al. 2021 Thompson et al. This content is distributed under the terms of the Creative Commons Attribution 4.0 International license.

Respondents (*n* = 105) indicated that they collect a number of different microbiological samples using lethal and noninvasive sampling of host species. Of the respondents, 41 (39.0%) indicated that they do not terminally sample hosts as part of any of their research projects. In contrast, 64 (61.0%) respondents indicated that their research does at least sometimes incorporate terminal sampling, including opportunistic sampling (e.g., accidental fatalities during capture) or work piggy-backed on other studies that included lethal sampling (e.g., abattoirs/slaughterhouses, culling of nuisance/pest species). Of the 64 respondents that include terminal sampling in at least some of their research, 36 (56.3%) respondents indicated using only “targeted intentional lethal sampling,” while the remaining respondents use a combination of terminal sampling methods. Importantly, of the 64 respondents who conducted terminal sampling, six did not collect microbiological samples that required terminal sampling of hosts (e.g., collected only nonlethal samples such as ectoparasites, excrement, etc.), and six collected only a single tissue type that required terminal sampling.

When participants were asked about practices related to archiving microbiological samples and host specimens, 75 of 104 respondents (72.1%) permanently archive collected microbiological samples, while only 51 of 107 respondents (47.7%) indicated that they permanently archive host species. Only 41 respondents (39.4%) report vouchering host specimens from which microbiological samples were collected. Of the 75 respondents that archived microbiological samples, 22 (29.3%) reported that they deposited their samples in biobanks/biorepositories and 26 (35.0%) deposited them in natural history collections, both of which typically provide permanent archiving of samples. However, most frequently, samples were stored in institutional laboratory space (60.0%), which is typically not accessible to the broader scientific community. When microbiological samples were reported not to be preserved, the majority of respondents indicated a lack of institutional infrastructure as the reason. These trends were generally reversed for the 51 respondents that reported permanently archiving host specimens in their studies, where 31 of the 51 (61.0%) indicated that their vouchers were deposited at natural history collections. For the 19 (30%) respondents who indicated that they include lethal sampling of hosts in their research but do not preserve voucher specimens, “no tradition of vouchering in my institute” and “no institutional infrastructure” were equally cited as the reason.

When participants were asked if they had ever collaborated with or deposited voucher specimens in a natural history collection, 44 of 105 respondents (41.9%) replied “yes” whereas 50 (47.6%) replied “no.” In addition, 11 (10.5%) indicated that they were unsure how to collaborate with a natural history collection. Of the 48 respondents who addressed the value of collecting host voucher specimens for studies of microorganisms, nearly all (93.8%) spoke to the benefits, often in terms of increased rigor and reproducibility of studies, with one reporting “The host is half of the equation in any host-parasite interaction, why wouldn’t we be vouchering the host?.” Three respondents did not see the value of vouchering, and one respondent expressed concerns about the biosafety of depositing specimens in a collection. Along this line, of the 42 respondents who wrote about their experiences working with natural history collections, 10 (24.4%) discussed problems, including sample types not being accepted, lost samples, and onerous logistical issues.

## ROLE OF NATURAL HISTORY COLLECTIONS IN HOST-PATHOGEN RESEARCH

### “Extending” the voucher specimen.

Biodiversity data archived in the natural history collections and biorepositories around the world continue to increase as novel questions arise and new research disciplines become aware of their existence (74). The past few decades have seen increasing collaborations between microbiologists and biodiversity scientists as the concept of the “holistic” or “extended” specimen has been adopted by more natural history collections (75). Under this paradigm, host and parasite specimens are collected simultaneously, along with associated cryopreserved tissues and microbiological samples, and all are linked through bioinformatics even if they are stored in different repositories (57, 76, 77). The merits of making these holistic collections are numerous as they naturally promote more integrated science (57, 64, 78). Integration of the “extended specimen” concept into host-pathogen research is now beginning as it is slowly becoming recognized as an essential part of zoonotic disease surveillance (69, 70, 72, 79).

Better integration of natural history collections in infectious disease studies would likely provide more clarity about the various roles of environmental change (e.g., biodiversity loss, climate change, land use change) (80) in the emergence of novel zoonoses (81). Such human-driven disturbances provide increased opportunities for pathogen transmission among host species (i.e., host switching from one wildlife species to others) resulting in heightened risk to public health, but risk assessment is difficult because we lack detailed information regarding naturally occurring host-pathogen relationships (78). Control of potentially zoonotic pathogens through targeted risk management (e.g., reducing contact between humans and reservoir hosts) is likely to be more effective than retroactive efforts aimed at eradicating zoonoses after they have spilled over into human populations (82). However, rigorous and successful prevention strategies require knowledge of historic and contemporary distributions, occurrence, and ecology and evolutionary history of host species in host-pathogen systems. Those critical assessments rely on information that largely resides in natural history collections (78, 83).

Below, we highlight three examples of successful integration of host-pathogen research and natural history collections in collaborative research programs focused on pathogens (i.e., yellow fever, hantaviruses, and helminths), as well as provide an example of a failed integration (i.e., ebolaviruses). Additional examples are provided in Table 1.

**TABLE 1:** Selected examples of host-pathogen studies that deposited and/or studied vouchered host specimens and microbiological samples in natural history collections

Pathogen	Disease	Host(s)	Natural history collection(s)	Reference(s)
Viruses				
Arenaviridae				
Guanarito virus	Venezuelan hemorrhagic fever	Rodents	American Museum of Natural History	155
Whitewater Arroyo virus	Hemorrhagic fever syndrome	Rodents	Natural Science Research Laboratory, Texas Tech University	156, 157
Coronaviridae				
Alpha- and betacoronaviruses	Pathogenicity unknown	Bats	Field Museum of Natural History	43
Middle East respiratory syndrome (MERS)-related betacoronavirus^*a*^	Pathogenicity unknown	Bats (*Neoromicia* spp.)	Ditsong National Museum of Natural History	42
Flaviviridae				
*Flavivirus* spp.	Yellow fever	Various	American Museum of Natural History, Smithsonian National Museum of Natural History, and Museu Nacional/Universidade Federal do Rio de Janeiro	86, 87, 90, 158–163
Filoviridae				
Ebolavirus	Ebola hemorrhagic fever	Vertebrates	Royal Museum of Central Africa and Museum of Southwestern Biology	110
Hantaviridae				
Laguna Negra virus	Hantavirus pulmonary syndrome	Rodents (Calomys laucha)	Field Museum of Natural History	164
Sin Nombre virus	Hantavirus pulmonary syndrome	Rodents	Museum of Southwestern Biology	94, 165
Anajatuba, Castelo dos Sonhos viruses	Hantavirus pulmonary syndrome	Rodents (*Oligoryzomys* spp.)	Museu Nacional/Universidade Federal do Rio de Janeiro	70, 96
Luteoviridae				
Barley yellow dwarf virus	Barley yellow dwarf	Grasses	University and Jepson Herbaria, University of California, Berkeley, and Center for Plant Diversity, University of California, Davis	166
Poxviridae				
Monkeypox virus	Monkeypox	Squirrels	American Museum of Natural History	167
Monkeypox virus	Monkeypox	Small mammals	Field Museum of Natural History	168

Parasites				
Arthropods				
Trombiculidae	Scrub typhus (*Rickettsia*)	Rodents	Smithsonian National Museum of Natural History	169
Syringophilidae	Ectoparasitic infestation	Birds	Natural History Department of Sarisske Museum	170
Trichodectidae	Ectoparasitic infestation	Gopher (*Cratogeomys* spp.)	Museum of Natural Science, Louisiana State University	171
Ixodoidea	Multiple tick-borne diseases	Mammals	Field Museum of Natural History	172, 173
Helminths				
Rictulariidae				
Paucipectines hymanae	Endoparasitic infection	Shrew opossum	Field Museum of Natural History	174
Spiruridae				
*Protospirura* spp.	Endoparasitic infection	Rodents	Muséum National d'Histoire Naturelle	175
Philometridae				
Clavinema marina	Endoparasitic infection	English sole (*Parophrys vetulus*)	Burke Museum of Natural History & Culture	108
Metastrongylidae				
*Skrjabingylus* spp.	Leptomeningitis	Skunks	Angelo State Natural History Collection	176
Dactylogyridae				
*Cichlidogyrus* spp.	Ectoparasitic infection	Cichlid fish	Royal Museum of Central Africa	104, 177
Schistosomatidae				
*Schistosoma* spp.	Schistosomiasis	Gastropods	Natural History Museum, London	178
Prokaryotes				
Trypanosomatidae				
Trypanosoma lewisi	Chagas disease	Rodents (*Rattus* spp.)	Natural History Museum, London, Museum of Zoology, University of Cambridge and Oxford University Museum of Natural History	179
Unicellular eukaryotes				
Plasmodiidae				
*Plasmodium* spp.	Malaria	Bats, primates, rodents	Field Museum of Natural History	180–183
*Hepatocystis* sp.	Malaria	Bats	Smithsonian National Museum of Natural History	184
*Nycteria* sp.	Malaria	Bats	Smithsonian National Museum of Natural History	185

Bacteria				
Leptospiraceae				
*Leptospira* spp.	Leptospirosis	Small mammals	Field Museum of Natural History	186, 187
Mycobacteriaceae				
Mycobacterium tuberculosis	Tuberculosis	*Bison* cf. *antiquus*	Carnegie Museum; University of Kansas Museum of Natural History	188
Yersiniaceae				
Yersinia pestis	Plague	Homo sapiens	State Collection for Anthropology and Palaeoanatomy	189
Yersinia pestis	Plague	Rodents	Museu Nacional/Universidade Federal do Rio de Janeiro	88

Fungi				
Peronosporaceae				
Phytophthora infestans	Potato blight	Potato	Various herbaria	190–192
Pseudeurotiaceae				
Pseudogymnoascus destructans	White-nose syndrome	Myotis bechsteinii	National Museum of Natural History	193
Meripilaceae				
Rigidoporus microporus	White rot disease	Rubber tree (Hevea brasiliensis)	Botanical Museum, Finnish Museum of Natural History & Botanical Museum, University of Helsinki	194

a MERS, Middle Eastern respiratory syndrome.

### Yellow fever.

One of the earliest efforts to employ comprehensive host and pathogen collection, vouchering, and data recording was the Yellow Fever Service, an organization maintained in the 1930s by the Brazilian Ministry of Education with the cooperation of the International Health Division of the Rockefeller Foundation (84, 85). The program was truly multidisciplinary, including fieldwork in collaboration with researchers from natural history collections to identify the animal reservoirs of the virus (86–88). As a result of the Yellow Fever Service, tens of thousands of voucher specimens, including amphibians, birds, mammals, and reptiles, were collected and deposited in natural history collections, and these have been employed in subsequent studies on systematics, ecology, evolution, and conservation biology of mammal species. Each sampled specimen was accompanied by extensive and standardized field notes (86), and the samples are accessible to researchers for further study. Among the major outcomes of this collaborative endeavor was the discovery of a sylvatic transmission cycle of the yellow fever virus (88, 89), eradication of Aedes aegypti in Brazil (90, 91) (albeit for just a few decades), and understanding primate population ecology and transmission risk of the yellow fever virus (92, 93).

The idea that voucher specimens provide scientific replicability, baseline data for temporal studies, correct taxonomic identification of host species, and material for future technological advances in host-pathogen research is clear in the seminal publication on results of the Yellow Fever Service by Gilmore (86):

The preservation of specimens for absolute identification in public health investigations is generally an acute problem, because trained field zoologists are often absent, and because the technique is a special one. With this in mind it is suggested that the documentation of every specimen by a protocol, with a few skins temporarily preserved by thorough drying in shape and the saving of even skulls of every different recognizable kind, designated consistently by the same common name, will enable identifications to be made at a later date with a certain amount of surety.

### Hantaviruses.

Hantaviruses provide one of the best examples of how to integrate natural history collections with infectious disease research (72, 94). Natural history collections have provided reference libraries for proactive and rapid identification of host species, in addition to temporal and spatial patterns, of new hantavirus strains. For instance, in 1993, pathobiologists were able to quickly identify the reservoir host of a novel hantavirus (Sin Nombre) responsible for a cluster of deaths in the Four Corners region of the southwestern United States by screening archived rodent specimens at the Museum of Southwestern Biology at the University of New Mexico and Natural Sciences Research Laboratory at Texas Tech University and determine that this novel virus had been present in host populations dating back at least as far as samples were available for screening (late 1970s) (94). New analyses or taxonomic revisions have also resulted in a revised understanding of the identities of many pathogen reservoirs subsequent to their original description. For example, recent systematic revisions of pygmy rice rats (genus *Oligoryzomys*) (i.e., 71, 95–98) have refined our understanding of this taxonomic group and have led to the recognition that many host reservoirs of hantaviruses have restricted geographic distributions. Changes resulting from studies of vouchered host specimens have led to the reassignment of host names for numerous hantaviruses of public health importance (e.g., Anajatuba, Bermejo-Neembucu, Castelo dos Sonhos, Choclo, and Maporal viruses) (99). In addition, the finding that hantaviruses occur in moles, shrews, and bats based on screening archived specimens from natural history collections has falsified the paradigm that this is solely a rodent-borne virus (100–102) and substantially changed our understanding of this family of pathogenic viruses.

### Helminths.

Mainly drawing from examples in fish parasitology, an inspection of host specimens originally not intended for parasitological research can serve a range of applications in the study of helminths (parasitic worms). Similar to research on other fish parasites that are unpredictable in prevalence or abundance (e.g., cymothoid isopods) (103), archived host specimens have later been used to describe new helminth species (104). For example, Kmentová et al. (105) characterized gill-infecting monogeneans of freshwater lates perch throughout Africa based on previously collected specimens. However, perhaps the most explicit advantage of voucher specimens to helminthological research is that natural history collections allow the inference of baseline data about parasite communities across various timescales. This potential for tracking disease emergence via shifts in pathogen occurrence and/or abundance and linking with environmental change was, to quote Harmon et al. (106), “hiding in plain sight”—collections of host specimens are indeed heavily underexplored for this purpose. Black (107) confirmed, from archived specimens of the lake trout (Salvelinus namaycush), the presence of the nematode Cystidicola stigmatura which has recently disappeared as a result of dwindling host abundance. Conversely, Howard et al. (108) demonstrated over a 9-decade time span the increased prevalence of the nematode Clavinema mariae infecting Puget Sound English sole (Parophrys vetulus). Invasion biology is another field where natural history collections provide a unique opportunity to establish a historical baseline for often understudied helminth communities. For example, Jorissen et al. (109) compared the monogenean gill parasites of historic and recent tilapia populations in Central Africa. This allowed distinguishing flatworms naturally infecting native fishes from those appearing only after cointroduction with nonnative tilapia species (and subsequent host switching).

### Ebolaviruses.

Ebolaviruses represent a group of pathogens where a lack of high-quality vouchered specimens across temporal scales has created a direct impediment to identifying putative reservoir hosts (110). Ranging from insects to antelopes (111), the identification of both reservoir host(s) and susceptible host populations remains largely unknown (112), although bats have been implicated as reservoir hosts for Reston ebolavirus (113), Bombali ebolavirus (114, 115), and Zaire ebolavirus (116, 117) but see Leendertz et al. (118) and Caron et al. (111). The ability to identify wildlife hosts of ebolaviruses is limited by a lack of centralized repositories of historical and modern voucher specimens, especially for mammal species, across the Afrotropics (119, 120).

Impediments in detecting ebolavirus hosts are related to both the distribution of the virus, most notably in the megadiverse and remote regions of the Afrotropics, and the highly unpredictable persistence of the virus in a host (116). Specifically, the prevalence of ebolaviruses circulating in populations of putative hosts, such as bats, can vary greatly across short periods of time (116) and is often lower (118) than prevalences observed in other viruses whose hosts have been identified more rapidly with the aid of voucher specimens (see the “Hantaviruses” section above). Furthermore, for mammals, the most targeted ebolavirus hosts thus far, the Afrotropics represents a hot spot of evolutionary diversity, with one recent study predicting at least 122 undiscovered species (121). Moreover, recent phylogenetic research using voucher specimens from natural history collections has indicated high levels of cryptic diversity in *Miniopterus* (122), a bat genus speculated to be a reservoir host for Zaire ebolavirus (123). Furthermore, studies on other filoviruses such as Marburg virus have found samples of different organs and body fluids (e.g., blood, colon, and oral-mucosa) collected from bats to be less effective than combined samples of liver and spleen in identifying positively infected bats (i.e., Rousettus aegyptiacus) using quantitative reverse transcription-PCR (Q-RT-PCR) (124, 125). Regardless of sample type collected and low prevalence rates of ebolavirus, limited sample sizes per host species from large and nonrandomized studies offer limited insight into our understanding of ebolaviruses (117).

A lack of historic and recent voucher specimens of mammals from the Afrotropics hinders effective predictions and identification of potential reservoir hosts of ebolaviruses in a number of ways. First, lack of historic material limits the ability to answer historical evolutionary questions regarding virus evolution, such as the consequences of viral population size on lineage extinctions through time (126). Second, a lack of systematically collected and holistic specimens of diverse groups of potential hosts such as bats, combined with myopic sampling of specific taxonomic groups (e.g., fruit bats) has resulted in biased sampling effort that underrepresents the true diversity of hosts (118). Third, a lack of archived samples available for screening pre- and postdisturbance by humans—a process thought to increase ebolavirus spillover between bats and humans (127)—limits our ability to understand the impacts of such processes on viral and host dynamics. Finally, although recent efforts to sample deceased mammals, a potentially important source of ebolavirus infection data (128), across parts of the Congo should be applauded (129), the lack of involvement of natural history collections in disease surveillance efforts is concerning (28).

## BEST PRACTICES FOR INTEGRATING NATURAL HISTORY COLLECTIONS IN HOST-PATHOGEN STUDIES

**FIG 1:**
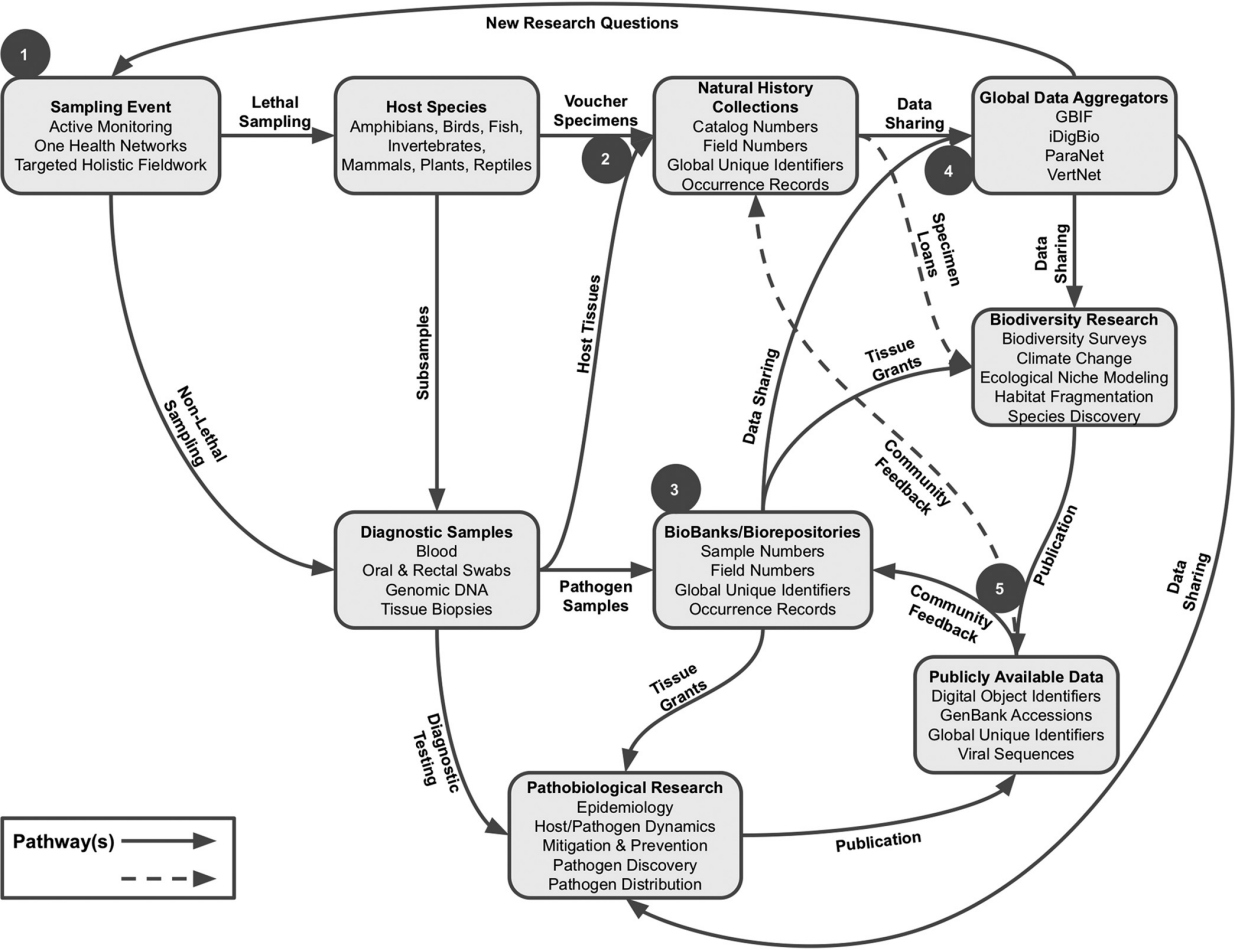
Workflow for integrating vouchering practices and natural history collections into host-pathogen research. Light-gray boxes provide descriptions for critical steps of the workflow, and arrows connecting the boxes indicate the pathway for the flow of specimens, samples, data, etc. Gray circles with white numbers represent steps described in the text.

Natural history collections have evolved to incorporate best practices for field collection, vouchering, sample preservation, and data sharing (74), creating a vast resource of globally distributed collections (e.g., 130–132). These best practices revolve around the voucher specimen as the common currency, which has resulted in a predictable workflow (Fig. 1). We highlight five critical steps in this workflow and provide recommendations for integrating vouchering techniques in infectious disease studies and fostering collaboration between natural history collections and the microbiological research community (see also Table S1 in the supplemental material for a nonexhaustive overview of collections with experience in host-pathogen research collaborations).

10.1128/mBio.02698-20.3 TABLE S1 Natural history collections with previous experience and/or interest in voucher-based host-pathogen studies. Abbreviations of natural history collections are as follows: AMNH, American Museum of Natural History; CTUA, Colección Teriológica, Universidad de Antioquia; FMNH, Field Museum of Natural History; GNM, Gothenburg Natural History Museum; LMUSP, Laboratório de Mamíferos da Escola Superior de Agricultura “Luiz de Queiroz,” Universidade de São Paulo; MECN, Museo del Instituto Nacional de Biodiversidad, Ecuador; MN, Museu Nacional, Universidade Federal do Rio de Janeiro; MNHN, Muséum national d’Histoire naturelle; MPEG, Museu Paraense Emilio Goeldi; MSB, Museum of Southwestern Biology, University of New Mexico; NHM, Natural History Museum, London; OMNH, Sam Noble Museum of Natural History, University of Oklahoma; PSUNHM, Princess Maha Chakri Sirindhorn Natural History Museum, Prince of Songkla University; QCAZ, Museo de Zoología de la Pontificia Universidad Católica del Ecuador; RMCA, Royal Museum for Central Africa; TTU, Natural Science Research Laboratory, Texas Tech University; UACH, Universidad Austral de Chile; UMMZ, University of Michigan Museum of Zoology. Download Table S1, DOCX file, 0.02 MB. Copyright © 2021 Thompson et al. 2021 Thompson et al. This content is distributed under the terms of the Creative Commons Attribution 4.0 International license.

### Step 1. Sampling event.

Sampling design is one of the most basic components of research. Given the complexity and nuances of wildlife research, early planning is critical, especially when working with pathogens. In addition to standard institutional animal care and biosafety protocols, local, state, and federal permits are often needed to sample and/or collect wildlife species. For international work, import and export permits, material transfer agreements, and compliance with the Nagoya Protocol on Access to Genetic Resources and the Fair and Equitable Sharing of Benefits Arising from their Utilization to the Convention on Biological Diversity may be necessary, as well as Convention on International Trade in Endangered Species (CITES) permits when dealing with threatened and endangered taxa. Natural history collections often have blanket permits and personnel (e.g., collection managers, registrars) that can assist in navigating wildlife research with relative ease. Integrating natural history collections early in sampling design would provide host-pathogen researchers the opportunity to leverage these resources, minimizing problems in downstream applications.

Broad temporal and spatial surveys of host taxa should have sample sizes sufficient to not only detect pathogens (which can occur at widely ranging prevalence rates, e.g., bat-borne viruses can range between 0 and 100% depending on sample type and viral family) (133) but also provide some level of natural history information about the target pathogen (94). For mammals, such as rodents, which tend to have large population sizes and high reproductive rates, targeted lethal sampling has been shown to not have significant adverse effects on population structure or density (134). For taxa that are threatened, long-lived, and/or have low reproductive rates (e.g., many bat and primate species), more limited collection efforts combined with nonlethal sampling methods (e.g., swabs, feces) may be necessary. Animals should be captured, handled, and euthanized following community standards for ethical care and use of vertebrates in research; for mammals, guidelines are provided by Sikes et al. (135).

Capture-sample-release surveys typically do not allow for repeatability or extension of the research (64). However, in some cases, nonlethal sampling may be all that is permitted. In these situations, sampling should still be maximized. Oropharyngeal and rectal swabs, feces, hair samples, ectoparasites, and wing punches or ear clips should be collected. Sample volume should also be maximized to provide sufficient material for multiple tests (i.e., isotopic analyses). Standard measurements and photographs of the host should also be taken to aid in species-level identification. However, measurements and photos are only complementary and alone are often not sufficient to provide unambiguous identification of hosts, particularly for diverse taxonomic groups with numerous cryptic species (e.g., rodents and bats) (120). Voucher specimens are the only reliable option to ensure repeatability and should be considered the gold standard in host-pathogen studies (70).

When hosts are euthanized or incidental deaths occur during nonlethal surveys, it is important to maximize the diversity of tissue types that are collected and preserved. Samples should be collected from the host immediately after death, and ideally, samples should be stored at ultracold temperatures (e.g., liquid nitrogen, dry ice) upon collection. Samples should be archived long-term in an ultracold (–80°C) freezer—but preferably in a vapor-phase nitrogen (–190°C) freezer—at accredited natural history collections and publicly available for use by qualified researchers (136). Alternatively, if tissue collection from hosts is not possible due to limitations in expertise, time, and/or funding, specimens can be preserved in formalin or alcohol (137). These low-cost options allow researchers the ability to obtain a verifiable voucher when euthanizing potential hosts and provide genetic material for species identification (138). An added benefit of formalin fixation of voucher specimens is that it is an approved method for inactivating viruses according to the Centers for Disease Control and Prevention (CDC) (139), thus rendering the deposited specimens noninfectious.

### Step 2. Depositing host specimens and tissue samples.

Natural history collections accession voucher specimens and associated tissue samples routinely as part of their mission. Although each institution has its own accession protocols (140), most natural history collections do not charge donors for depositing voucher specimens and tissue samples, but early communication prior to a collection event is recommended in order to address any logistical or financial hurdles. All deposits of voucher specimens and tissues require documentation showing the legal framework under which they were obtained, as well as any data associated with the deposited materials to confirm their legal status.

To facilitate accessibility to the broader scientific community, researchers should shift samples from their personal laboratories to permanent repositories such as natural history collections when samples are no longer needed for the original study. Alternatively, most natural history collections will place temporary embargoes on deposited specimens and samples to allow for the original study to continue while ensuring they are permanently and safely archived. Of course, if a researcher is engaged collaboratively with a curator or collection manager, this process will be part of project development and integrated into the study design.

Full metadata should be included with each specimen and sample. The most basic metadata minimally required by all natural history collections are collection date, geographic location (preferably including both a textual locality and GPS coordinates), collector’s name, and some level of taxonomic identification. In addition, depending on the disposition of the specimens, vouchers often include taxon-specific measurement and life history data. Even when collecting nonlethal samples, metadata should be recorded and shared with the receiving institution when depositing samples.

Last, the receiving institution will formally accession the deposited specimens and/or samples in order to transfer custody to the natural history collection (141). Although custody of the voucher specimens and samples has changed, an embargo can remain in place to allow time for publication and the development of additional projects. This time period also allows collection managers and curators to verify species identifications, curate metadata, and assign formal catalog numbers to the voucher specimens and/or samples (e.g., MSB:Mamm:89863). For researchers engaged in host-pathogen research, this process provides verification of the host vouchers based on current taxonomy, in addition to assignment of a globally unique identifier (GUID) associated with the specimen. GUIDs allow the scientific community the opportunity to digitally curate voucher specimens and link data repositories, literature, and other resources to the specimen (142). Voucher specimens and samples are of no value if they are not given unambiguous unique numbers, thereby preventing data linkage.

### Step 3. Archiving microbiological samples.

Archiving microbiological samples from host species that may be infectious requires considering the biosafety capabilities of the receiving collections. Researchers should inform the receiving natural history collection of pathogens detected in deposited samples as well as any treatments to the vouchers and/or tissue samples to deactivate specific pathogens of concern. Concomitantly, the natural history collection should inform the donor of any limitations in their ability to receive potentially pathogenic samples. However, many natural history collections maintain biorepositories to complement their vouchered host collections (143) and utilize best practices for sample curation and personnel safety (136, 144). Specifically, museum personnel should be trained in biosafety protocols for handling pathogenic samples and implementation of increased security measures, such as restricted access to designated areas where pathogenic samples are stored. In addition, if future research is to be conducted using pathogenic samples, many natural history collections are equipped with biosafety level 1 and/or 2 laboratory space. Under certain circumstances, in particular research on designated CDC Select Agents, samples need to be deposited in a biorepository or biobank with higher biosafety level capabilities (145).

Similar to vouchering host specimens and tissues, pathogenic samples should be well-documented with appropriate permits and metadata. Ideally, the metadata should include all data associated with the host species plus pathogen-specific information. When possible, a molecular identification (e.g., nucleic acid sequence) for both the host (symbiotype) and pathogen should be included so that their identities can readily be placed on the Tree of Life and provide a basis for identifying sister species that may serve as potential hosts for related pathogens. GUIDs also should be assigned to microbiological samples and related back to the host specimen record.

### Step 4. Specimen access and data sharing.

The archiving institution should maintain a relational collections database (e.g., Arctos, EMu, Specify), which should be linked to biodiversity data aggregators (e.g., GBIF, iDigBio, FishBase, and VertNet) and data repositories (e.g., GenBank, IsoBank, MorphoSource) through reciprocal linkages or via an integrated publishing toolkit (146). By assigning GUIDs during the archiving process, researchers can connect these typically disparate data sets into a single aggregated view of associated vouchered specimens and samples through online searchable databases. This allows all derivative data to be tied back to the original vouchered specimen and/or sample (147), allowing for a more integrated approach to host-pathogen research (72). Any updates made to the vouchered specimen (e.g., species identification, georeferencing, new data sets) would be updated through the network of aggregators and repositories and be made available to the end user. In addition, this makes the vouchers discoverable and available to researchers both remotely online and physically through collection visits and loans.

### Step 5. Publication and data repositories.

Citing accession numbers of vouchered specimens and/or samples in publications is essential to link data sets back to natural history collections and biorepositories. A full list of specimens examined and the associated pathogen screening results should be included in publications or minimally available as supplemental material. The individual host specimen from which the novel pathogen is sequenced and/or isolated, and then described, should also be formally designated a symbiotype, along with other specimens from the type series containing the novel pathogen (i.e., symbioparatypes) (148). Symbiotype identity should be confirmed with a DNA sequence (e.g., cytochrome *b* for mammals) and deposited in National Center for Biotechnology Information (NCBI) BioSample. The biosample attribute “voucher_specimen” is linked to the species name in the biosample. If you are submitting a pathogen sequence (including a full genome), a new attribute, host_specimen_voucher, should be used to store the museum catalog number of host specimen information for SARS-CoV-2 (or any other pathogen). For GenBank submissions, it is recommended to link to a registered BioSample (see reference 149). When naming newly discovered pathogens, the symbiotype catalog number should be included in the name (e.g., Camp Ripley virus, MSB:Mamm:89863) to facilitate linkage between host and pathogen. This same approach should be used when depositing data sets on Dryad and other publicly available data repositories, allowing for linkages back to the physical voucher specimen (147).

## CONCLUSIONS

The COVID-19 pandemic has exposed many weaknesses in the current public health approach to zoonotic spillover events and has reinforced the need for shifting to an integrated One Health approach for addressing EIDs (150). In particular, the critical importance of identifying a definitive host of SARS-CoV-2 (4, 11) coupled with the unprecedented potential of current molecular techniques to reinvestigate historic material archived in natural history collections (e.g., human immunodeficiency virus 1 [HIV-1] genome recovered from samples collected in 1966, prior to the discovery of acquired immunodeficiency syndrome [AIDS]) (151) highlights the need for a more integrated approach to surveillance and mitigation of EIDs (70). Vouchering host specimens and associated tissue samples in natural history collections is one of the most effective means of overcoming this hurdle.

To meet their promise of substantially contributing to infectious disease studies, however, natural history collections must grow exponentially in the coming decades (152, 153) and invest in methodology and infrastructure development which meets best practices to maximize the utility of deposited specimens and samples for future research projects (57, 64, 70, 136). When properly cared for (140), natural history collections can last centuries, providing a physical record for current and future pandemics (148). By vouchering and depositing host specimens, associated tissue, and microbiological samples in natural history collections, the host-pathogen research community will contribute to future research endeavors and mobilize a large community of biodiversity scientists into One Health networks (154).
