# Supplementary material for: Preserve a Voucher Specimen! The Critical Need for Integrating Natural History Collections in Infectious Disease Studies
Source: mBio. 2021 Jan 12;12(1):e02698-20. doi: 10.1128/mBio.02698-20 (PMC7844540; doi:10.1128/mBio.02698-20)
Supplement: TEXT S1 [file mBio.02698-20-s0001.docx]

**SUPPLEMENT S1.** Microbiology sampling methods and museums – a human survey

**Survey Methodology and Institutional Review Board (IRB) approval**

The survey was reviewed by the IRB at Bucknell University in Lewisburg, Pennsylvania, USA, and determined to be “exempt from further review” on 27 May 2020 (IRB protocol # 1920-126). The survey was hosted on the Qualtrics© XM survey platform and, beginning 30 June 2020, was distributed widely to members of diverse research networks and on social media platforms. Data were downloaded for analysis on 5 August 2020. The survey was attempted a total of 183 times and fully completed by 112 users. Analysis was performed on completed surveys only, with one non-scientist excluded from analysis, for a total of 109 survey respondents. Raw data is available in DATA S1.

**Survey Information and Consent Form**

Microbiology Sampling Methods and Museums - Informed Consent, Bucknell University

The [Natural Science Collections COVID-19 Task Force](https://cetaf.org/covid19-taf-communities-taking-action) (composed of researchers involved with natural history collections and/or biodiversity sciences) set as one of its aims to develop guidelines for linking host and microorganism/parasite samples and data. For this, we are requesting your participation in a brief questionnaire on common practices used by microbiologists for collecting and preserving study organisms (viral and otherwise) and archiving (collecting a voucher specimen) of the host organism.

We estimate that this survey will take less than 15 minutes and anticipate receiving responses from ~500 participants. A series of multiple choice and free choice questions will be asked. The survey results will benefit society by informing scientists about the common practices of microbiologists with regards to archiving pathogen host data, samples, and specimens. Subjects will benefit by enhanced awareness of possible research collaborations and practices. Improved knowledge will facilitate greater connections and collaborations between those who study pathogens and those who study their hosts, informing emerging infectious disease risk. There are no foreseeable risks or discomforts to you as a participant. Your participation is voluntary, refusal to participate or discontinuing participation at any time will involve no penalty or loss of benefits to which you are otherwise entitled. All data are being collected anonymously and confidentially. You can reach the principal investigator for questions about this research survey (DeeAnn Reeder, Department of Biology, Bucknell University, at 570.577-1208 or [deeann.reeder@bucknell.edu](mailto:deeann.reeder@bucknell.edu)). Questions or concerns about research subjects' rights and research-related injury can be directed to Matthew Slater, the Chair of the IRB, at 570.577.2767 or [matthew.slater@bucknell.edu](mailto:matthew.slater@bucknell.edu).

By clicking "I agree" below, I affirm my participation in this study.

- Yes, I agree
- No, I do not agree

**Survey Questions**

Instructions: Please thoroughly read each question before answering. Note that some questions allow for the selection of multiple answers.

**1.** **With what type(s) of institution are you formally affiliated?**  (Select all that apply.)

- University
- Government research institute
- Independent research institute
- Government agency
- Public health laboratory
- Non-governmental organization
- Non-profit organization
- Hospital or clinic
- Natural history museum
- Other, please specify
- Prefer not to respond

**2. Please select the country of your primary affiliated institution** (drop down menu).

**3. Highest terminal degree**:

- Bachelor's degree
- Master's degree
- MD/DO
- DVM
- PhD
- MD/DO and PhD
- DVM and PhD
- Other, please specify: ________________________________________________
- Prefer not to respond

**3a. Year your highest terminal degree was awarded** (drop down menu).

**4. What is/are your field(s) of research?**  (Select all that apply.)

- Biomedical/clinical sciences (human)
- Veterinary science
- Systematics & evolutionary biology
- Virology or microbiology
- Ecology
- Public health policy or economics
- Social or behavioral sciences
- Other, please specify: ________________________________________________
- Prefer not to respond

**5. Which microorganism(s) are the focus of your research?**  (Select all that apply.)

- Viruses
- Bacteria
- Fungi (e.g., yeasts, molds)
- Protozoans
- Helminths (e.g., monogeneans, digeneans, tapeworms, acanthocephalans, nematodes, leeches)
- Other, please specify: ________________________________________________
- Prefer not to respond

**6. Does your research include lethal (terminal) sampling of hosts?  If different projects have different answers, select all that apply.**

- Yes, but only opportunistically (e.g., accidental fatalities during capture)
- Yes, but only because hosts were already being euthanized for other reasons (e.g., abattoirs/slaughterhouses, culling of nuisance/pest species)
- Yes, my research included targeted intentional lethal sampling
- No
- Prefer not to respond

**7. As part of your research, what biological samples do you collect to study the selected microorganism(s)?** (Select all that apply.)

- Liver
- Lung
- Muscle
- Stomach
- Intestine
- Spleen
- Kidney
- Brain
- Lymph nodes
- Oral swabs
- Rectal swabs
- Nasal swabs
- Excrement
- Whole blood
- Serum or plasma
- Wing biopsy punches
- Ectoparasites
- Hairs
- Mucus
- Ear clippings
- Toe clippings
- Environmental samples (soil, water, etc.)
- Other, please specify:________________________________________________
- Prefer not to respond

**8. If you collect biological samples, which fixative or storage medium do you use?**  (Select all that apply.)

- Undenatured ethanol
- Denatured ethanol
- Isopropyl alcohol
- Formaldehyde
- Formalin
- RNALater
- DNA/RNA shield
- Viral transport media (VTM)
- TRIzol/TRIreagent
- Lysis buffer
- Filter paper (e.g., FTA cards, Nobuto strips)
- Desiccation
- Liquid nitrogen (flash frozen with no fixative or storage medium)
- Another type of cold chain, please specify:________________________________________________
- Other, please specify:________________________________________________
- Prefer not to respond

**9. If you research requires fieldwork, what cold chain practices do you use in the field setting?**(Select all that apply.)

- Ultra-cold storage (-80 °C) (e.g. liquid nitrogen tank, cryoshipper, dry ice)
- Freezer (-20 °C) (e.g. portable freezer, insulated container with ice)
- Refrigerator (4 °C) (e.g. cooler with gel packs)
- Samples kept at room temperature temporarily
- Long-term storage of samples at room temperature
- Prefer not to respond

**10. In which country(ies) is your fieldwork conducted or from which do you obtain**

**samples?**  (Select all that apply; drop down menu.)

**11. Do you archive (voucher) any collected microbiological samples permanently?**

- Yes
- No
- Prefer not to respond

**11a. If yes, where do you deposit the samples?**  (Select all that apply.)

- Institutional laboratory space
- Natural history museum (in country)
- Natural history museum (international)
- Biobank/biorepository
- Other, please specify: ________________________________________________

**11b. If no, why do you not archive (voucher) any collected microbiological samples permanently?**

- Funding agency requires the destruction of samples upon project completion
- No institutional infrastructure to store samples in country
- No institutional infrastructure to store samples in the country where samples were screened
- Other, please specify:________________________________________________

**12. Do you archive (voucher) the sampled hosts permanently?**

- Yes
- No

**12a. If yes, how are the specimens (host) preserved?**  (Select all that apply.)

- Fluid preserved (in formalin, ethanol, etc.)
- Skin and skull and/or skeleton preparation (for vertebrate hosts only)
- Other, please specify:________________________________________________

**12b. If yes, where do you deposit the voucher specimens?**  (Select all that apply.)

- Natural history museum (in country - in country of origin of voucher)
- Natural history museum (international - outside of country of origin of sample)
- Teaching collection
- Institutional storage space
- Personal collection
- Other, please specify: ________________________________________________

**12c. If no, why do you not collect voucher specimens?**   (Select all that apply.)

- Not allowed by permitting agencies
- No institutional infrastructure to store voucher specimens in country
- No institutional infrastructure to store voucher specimens in the country where the samples are analyzed
- No export/import permit
- There is no tradition of vouchering in my research field
- There is no tradition of vouchering in my institute
- Ethical considerations related to invasive or lethal sampling
- Ethical considerations related to accessing genetic resources
- Time constraints (e.g., lack of time)
- Lack of expertise
- Other, please specify:________________________________________________

1**3. What value, if any, do you think collecting a voucher specimen of the host adds to studies of microorganisms?**  (Skip this question if you prefer not to respond.)

________________________________________________________________

________________________________________________________________

**14. Have you ever collaborated with or deposited voucher specimens in a natural**

**history museum collection?**

- Yes
- No
- Not sure how to collaborate with a natural history museum collection

**15. Please comment on your experience(s) with collaborating or attempts to collaborate with a natural history museum collection.**  (Skip this question if you prefer not to respond.)

________________________________________________________________

________________________________________________________________

**16. Have you experienced barriers to publishing voucher numbers or more details in submission at journals or NCBI?**  Please explain your answer.  (Skip this question if you prefer not to respond.)

________________________________________________________________

________________________________________________________________

**Summarized survey results**

After excluding subjects that did not finish the survey or were not scientists, a total of 109 surveys were analyzed (see DATA S1 for raw survey results). Most respondents (73 of 109; 67.0%) were affiliated with a university; three of these were also associated with governmental research institutes or agencies, as were another 26 (23.9%) respondents. Remaining respondents (19 in total selected more than one type of affiliation) were associated with natural history museums (8; 7.3%), independent research institutions (8; 7.3%), hospitals or clinics (4; 3.7%), and public health laboratories (3 or 2.8%). Survey participants were from all continents except Antarctica, with heavy representation from Europe (42; 43.3%) and North America (24; 24.7%), followed by South America (15; 15.5%), Africa (11; 11.3%), Australia and New Zealand (3; 3.1%), and Asia (2; 2.1%); and 12 participants did not indicate the country of their current institution. Respondents reported obtaining samples from an even broader global distribution - 152 total countries (77.9% of all currently recognized countries!). Across 104 respondents, 49 (47.1%) indicated that they conduct fieldwork or obtain samples from multiple countries including 28 respondents reported using samples collected in the United States and Canada, 127 in Europe, 72 in Latin America, 16 in Australasia, 158 in Africa, 64 in Asia, and 16 in the Caribbean.

The vast majority of respondents hold a Ph.D. (81; 74.3%), 11 of these held dual degrees (e.g., MD, DVM), 23 (21.1%) identified their terminal degree to be at the Masters level, and five (4.6%) at the Bachelors level. Although 19 respondents did not answer when asked the year of their terminal degree, the survey responses were generally biased towards presumably younger scientists, with a total of 54 (49.5%) receiving this degree in the past decade and 72 total (66.1%) in the past 20 years. Participants were asked to classify their field(s) of research, 42 of the 108 (38.9%) respondents indicated two or more disciplines, reflecting the interdisciplinary nature of much scientific research today. The most frequently selected discipline was “Systematics and evolutionary biology” (45; 41.3%), followed by “Ecology” (42; 38.5%); 16 selected both disciplines, and often additional ones. “Virology or microbiology” as well as “Parasitology” were also frequent choices (37 and 13 respondents, or 33.9% and 11.9%), as were “Veterinary science” (15; 13.8%) and “Biomedical/clinical sciences (human)” (13; 11.9%).

Not surprisingly given the diversity of our respondents, many (44; 41.5%) indicated studying more than one taxonomic group of microorganisms. Helminths (e.g., monogeneans, digeneans, tapeworms, acanthocephalans, nematodes, leeches, etc.) was the most frequently cited focal taxa (47; 43.1%), followed by bacteria (40; 37.7%), viruses (25; 23.6%), protozoans (27; 25.5%), and fungi (17; 16.0%). Additional answers included “parasites” (3), Apicomplexa (1), and various vectors (snails (1), insects (2), rodents (1), bats (1), and “vertebrates” (1).

When asked to describe whether their research included lethal (terminal) sampling of vertebrate host species, respondents were directed to select multiple answers if different projects had different sampling protocols and/or permissions. Nearly half of all respondents (47 of 105; 44.8%) indicated that they do not terminally sample host species. Of these 47 respondents, 41 (87.2%) selected no other answer, thus all of their studies explicitly do not include terminal host sampling, even when host animals accidentally die during capture and sampling. Most respondents (64; 58.7%) indicated that their research does include terminal sampling. Thirteen of these 64 (20.3%) indicated that their terminal host sampling was not “targeted intentional lethal sampling”, it was either opportunistic (e.g., accidental fatalities) or piggy-backed on other studies that included lethal sampling efforts (e.g., culling of nuisance species, abattoirs/slaughterhouses). Thus, of the 64 respondents that included terminal sampling of host species, 51 (79.7%) collected their samples using protocols that explicitly “targeted intentional lethal sampling”. Roughly half of 107 respondents (51; 47.7%) indicated that they permanently archive these host vouchers, with a majority of the 51 respondents (31; 60.8%) depositing voucher specimens at natural history collections. For the 56 (of 107) respondents who do not preserve host vouchers, “no tradition of vouchering in my institute” and “no institutional infrastructure” were equally cited.

Respondents indicated collecting a number of different types of lethal and non-invasive samples, which mapped nearly perfectly onto our three cohorts of scientists: (1) those who reported never terminally sampling, (2) those who only terminally sampled opportunistically or by piggy-backing on another study, and (3) those who explicitly targeted lethal sampling. Importantly, of the 51 respondents who explicitly “targeted intentional lethal sampling”, six did not indicate the collection of any tissues or other biological samples that require terminal sampling, and six collected only a single tissue type that required terminal sampling. Although ultra-cold (-80°C) storage (e.g. liquid nitrogen tank, cryoshipper, dry ice) during field sampling is ideal for preserving most types of microbiological samples, only 39 of 106 (29.1%) respondents reported using ultra-cold storage during fieldwork. Other storage methods (e.g., FTA cards) and storage media (e.g., DNA/RNA Shield) were also frequently used, and can facilitate sampling when cold storage is not possible logistically or prohibitively expensive.

When participants were asked about practices related to archiving microbiological samples, 75 of 104 respondents (72.1%) reported permanently archiving collected microbiological samples but only 41 of 104 (39.4%) respondents report vouchering of host specimens from which these microbiological samples were collected. Microbiological samples were reported to be archived permanently in biobanks/biorepositories (22 of 75; 29.3%), natural history museums (26; 35.0%), but most frequently in institutional laboratory space (45; 60%). When microbiological samples were reported not to be preserved, the vast majority of respondents indicated “a lack of institutional infrastructure”. These trends were generally reversed for the 51 respondents that reported permanently archiving host specimens in their studies, where 31 (60.8%) indicated that their vouchers were deposited at natural history collections.

When asked if they had *ever* collaborated with or deposited voucher specimens in a natural history museum collection, 44 of 105 (41.9%) respondents said “Yes”, 50 (47.6%) said “No”, and 11 (10.5%) indicated that they were “Unsure” how to do this. Of the 48 respondents who addressed the value of collecting host voucher specimens for studies of microorganisms, nearly all (45; 93.8%) spoke to the benefits, often in terms of increased rigor and reproducibility of studies, with one reporting “The host is half of the equation in any host-parasite interaction, why wouldn't we be vouchering the host?”. Three participants did not see the value and one questioned the safety of depositing specimens in a museum. Along this line, of the 42 respondents who wrote about their experiences working with museums, 10 (24.4%) respondents discussed problems and concerns, from sample type not being accepted, to samples being lost, to onerous logistical and bureaucratic issues. Lastly, respondents were asked if they experienced any barriers to publishing voucher numbers in scientific journals or to the National Center for Biotechnology Information (NCBI). Of the 31 participants that had experience publishing voucher numbers, only 3 (9.7%) reported negative experiences (e.g., museums taking too long in reporting archive numbers of deposited voucher specimens).
