# Supplementary material for: Preserve a Voucher Specimen! The Critical Need for Integrating Natural History Collections in Infectious Disease Studies
Source: mBio. 2021 Jan 12;12(1):e02698-20. doi: 10.1128/mBio.02698-20 (PMC7844540; doi:10.1128/mBio.02698-20)
Supplement: TABLE S1 [file mBio.02698-20-st001.docx]

**TABLE S1.** Natural history collections with previous experience and/or interest in voucher-based host-pathogen studies. Abbreviations of natural history collections are as follows: **AMNH** - American Museum of Natural History; **CTUA** - Colección Teriológica, Universidad de Antioquia; **FMNH** - Field Museum of Natural History; **GNM** - Gothenburg Natural History Museum; **LMUSP** - Laboratório de Mamíferos da Escola Superior de Agricultura "Luiz de Queiroz", Universidade de São Paulo; **MECN** - Museo del Instituto Nacional de Biodiversidad, Ecuador; **MN** - Museu Nacional, Universidade Federal do Rio de Janeiro; **MNHN** - Muséum national d'Histoire naturelle; **MPEG** - Museu Paraense Emilio Goeldi; **MSB** - Museum of Southwestern Biology, University of New Mexico; **NHM** - Natural History Museum, London; **OMNH** - Sam Noble Museum of Natural History, University of Oklahoma; **PSUNHM** - Princess Maha Chakri Sirindhorn Natural History Museum, Prince of Songkla University; **QCAZ** - Museo de Zoología de la Pontificia Universidad Católica del Ecuador; **RMCA** - Royal Museum for Central Africa; **TTU** - Natural Science Research Laboratory, Texas Tech University; **UACH** - Universidad Austral de Chile; and **UMMZ** - University of Michigan Museum of Zoology.

| **Collection** | **Description** | **Tissue Holdings** | **Host-Pathogen Experience** |
| --- | --- | --- | --- |
| **AMNH**  Mammal  Collection | **Size**: ~280,000 cataloged morphological specimens (skins, skulls, skeletons, fluid-preserved material); including >1200 name-bearing types (holotypes, syntypes, lectotypes, neotypes).  **Composition:** Includes specimens representing 100% of 28 Recent orders, 96% of 153 Recent families, ca. 50% of 1229 Recent genera, and ca. 60% of 5416 Recent species; Includes specimens of 353 taxa listed in CITES Appendix 1 and/or as Endangered by IUCN. Worldwide geographic coverage - collections from Australia/New Guinea (>29,000 specimens), central Asia (>12,000), equatorial Africa (>29,000) and South America (>50,000).  **Strengths**: Collections of marsupials, bats, rodents, primates, carnivores, insectivores, and ungulates are among the most comprehensive in the world. | **Size:** ~4,000 tissue samples maintained in the Ambrose Monell Cryo Collection.  **Composition:** All samples maintained in -80^o^C facility, but many initially preserved in ethanol, RNA*later*, or other preservatives.  **Strengths:** Small mammals, especially bats, Neotropical taxa particularly well represented. | - Lyme Disease - Rabies - Venezuelan Hemorrhagic Fever - Yellow Fever |
| **CTUA**  Mammal Collection | **Size**: ~5,800 specimens representing ~250 species  **Composition:** Skin, skull, postcranial skeletal, fluid-preserved whole organisms, tissues and ectoparasites in ethilic OH.  **Strengths:** Strong representation of NW Colombian mammals; mostly bats, rodents and opossums. Species ID’s for over 95% of our holdings. | **Size**: 1,000-1,200 samples  **Composition:** The majority of the samples are preserved in ethanol.  **Strengths:** No information provided. | - Hantavirus - Leptospira - Rickettsia - Robovirus |
| **FMNH**  Mammal  Collection | **Size:** >243,500 voucher specimens representing >2,500 species including >575 name-bearing types.  **Composition:** Skin, skull, post-cranial skeletal, fluid-preserved whole organisms, frozen tissue (muscle, heart, kidney, liver, spleen, blood, ear clips), auxiliary collections (embryos, GI tracts, brains, baccula), endocasts, ecto- and endoparasites.  **Strengths:** All orders and all but one extant family (the monotypic bat family Craseonycteridae) and is worldwide in scope, with strong geographic representation from Africa (>80,000), Asia (>50,000), North America (>51,000), and South America (>47,000). | **Size:** >72,000 tissue samples  **Composition:** All samples maintained in liquid nitrogen cryo-facility with many initially preserved in ethanol or DMSO but with multiple field expeditions using liquid nitrogen as initial preservation.  **Strengths:** Small mammals, especially bats and rodents, with strong representation from the Afrotropical, Indo-Malaysian, Madagascan (esp. afrosoricids), Neotropical, and Patagonian Realms. | - Bartonella - Ectoparasites - Endoparasites - Coronavirus - Leptospira - Hantavirus - Malaria - Monkeypox - Rift valley fever |
| **LMUSP**  Mammal  Collection | **Size**: ~2,500 specimens  **Composition:** Skin, skull, post-cranial skeletal, fluid-preserved whole organisms, frozen tissue (liver and muscle)  **Strengths:** Collections of marsupials, bats and rodents from Western (Japurá, Iça, Madeira, Abacaxis) and Eastern (Xingu) Brazilian Amazon, as well as specimens from Atlantic Forest and Cerrado. | **Size**: ~2,500 samples of 2,000 specimens.  **Composition:** mostly alcohol preserved samples kept in -20^o^C freezer.  **Strengths:** samples from previously unsampled species and specimens. | - Spotted Fever |
| **MECN**  Mammal  Collection | **Size**: ~ 6,200 specimens  representing ~ 360 species  **Composition:** skin, skull, post-cranial skeletal, fluid-preserved whole organisms, digestives, tissue samples, ectoparasites and endoparasites  **Strengths:** No information provided. | **Size**: ~ 2,000 specimens  **Composition:** The majority of the samples are frozen, but the collection also contains samples in ethanol.  **Strengths:** No information provided. | - Studies are being carried out in collaboration with foreign partners (Universidad de La Plata) and with other Ecuadorian like University Yachay Tech University. |
| **MNHN**  Mammal Collection | **Size:** >130,000 specimens (skins, skulls, skeletons, fluid-preserved material) including ~1,500 name-bearing types  **Composition: :** Skin, skull, post-cranial skeletal, fluid-preserved whole organisms, tissues in alcohol, frozen tissues, tissues in RNA-later, karyotype test slides  **Strengths:** All orders represented, from all continents. Strong geographic representation from Africa, and to a lesser extent from Europe. Large series for some species. | **Size and composition**: >33,000 samples preserved in ethanol, 1,200 cryotubes with explants or cell suspensions preserved at -80°C, 280 samples preserved in RNA*later.*  **Strengths:** mostly African small mammals (rodents, shrews and bats) | - Hantavirus - Nematodes |
| **MN**  Mammal Collection | **Size**: ~100,000 specimens  **Composition:** Skin, skull, postcranial skeleton, fluid-preserved whole organisms, frozen tissue (liver and muscle).  **Strengths:** All orders from Brazil represented, large samples of Chiroptera, Primates, Rodentia, Carnivora, Xenarthra, and Didelphimorphia. | **Size**: ~ 15,600 samples of ~ 7,000 specimens.  **Composition:** Ethanol preserved samples, kept in conventional and ultra- freezers (-20 C° to -40 C°).  **Strengths:** Rodentia, Didelphimorphia, Primates, and Chiroptera | - Bartonella - Coxiella - Ectoparasites - Hantavirus - Rickettsia - Nematodes - Yellow fever |
| **MPEG**  Mammal Collection | **Size**: ~46,000 specimens  **Composition:** Skin, skull, postcranial skeleton, fluid-preserved whole organisms, frozen tissue (liver and muscle)  **Strengths:** All orders from Brazil represented, with focus on Chiroptera, Primates, Rodentia, and Didelphimorphia from Brazilian Amazonia (mainly eastern region). | **Size**: ~ 1,900 samples of ~ 1,900 specimens  **Composition:** Ethanol preserved samples, kept in conventional freezer (-20 C°).  **Strengths:** mainly samples from  Rodentia, Didelphimorphia, Primates, and Chiroptera (in this order) | - Ongoing research on groups of medical or veterinary importance (researchers from UFPA, UEPA, and Instituto Evandro Chagas), being the MPEG final repository of the host organisms. |
| **MSB**  Mammal  Collection | **Size:** >327,000 voucher specimens representing >1,750 species  **Composition:** Skin, skull, post-cranial skeletal, fluid-preserved whole organisms, frozen tissue (heart, kidney, liver, lung, spleen, muscle, blood, ear clips), cell suspensions, karyotype test slides, ecto- and endoparasites  **Strengths:** Strong holdings from the western United States, Beringia, Central Asia, and Latin America. The Division of Mammals holds traditional type specimens representing 85 taxa (68 with tissue held in DGR), symbiotypes of 179 ecto- and endoparasites, and 32 symbiotypes of 18 novel hantaviruses. | **Size:** >584,000 samples from >290,000 individual organisms.  **Composition:** Archives cryogenically preserved samples of animal tissues, whole organisms such as embryos and parasites, and purified DNA and RNA  **Strengths:** No information provided. | - Bartonella - Chagas - Ebola - Hantaviruses - Parasites |
| **NHM**  Schistosomiasis Collection | **Size**: >500,000 individual specimens  **Composition:** larval schistosomes (and a small number of other trematodes), adult schistosomes, snail intermediate hosts (primarily *Biomphalaria* and *Bulinus*)  **Strengths:** Significant temporal and spatial resolution, large, multiple redundancy in collections allows study of population-level genetic diversity of schistosomes, causing a neglected tropical disease endemic in 78 countries. | **Size**: >500,000 larval trematodes (primarily *Schistosoma mansoni* and *Schistosoma haematobium*). ~200,000 snails from ~110 genera (primarily schistosome intermediate hosts), ~20,000 adult schistosome worms  **Composition:** Larval trematodes are preserved on Whatman FTA cards. ~75,000 snails preserved in ethanol, >10,000 DNA and tissue vouchers, remainder - legacy collections: in industrial methylated spirit, adult schistosomes cryopreserved in vapour-phase liquid nitrogen  **Strengths:** Collected as a biorepository to support research into schistosomiasis; temporal and geographical resolution of collections. | - Detection/Diagnostics - Genomics/evolution - Population genetics/mapping |
| **OMNH**  Mammal  Collection | **Size:** >67,000 cataloged specimens representing ~900 species; including 2 holotypes, paratypes of 9 species, 9 symbiotypes and 17 parasymbiotypes.  **Composition:** Skin, skull, skeletons, fluid-preserved whole organisms  **Strengths:** Rodentia and Chiroptera with significant holdings from Argentina and Mexico, as well as other Neotropical countries, Yellowstone National Park, and all 50 US states. | **Size:** >31,000 tissue samples (heart, kidney, liver, muscle, spleen) maintained in the Oklahoma Collection of Genomic Resources.  **Composition:** All samples maintained in liquid nitrogen cryo-facility with initial field preparation including flash frozen, ethanol, lysis buffer, and DMSO.  **Strengths:** Small mammals, especially rodents, shrews and bats, with strong representation from the Neotropics. | - Ectoparasites - Endoparasites - Hantavirus - Paramyxovirus - Rabies |
| **PSUNHM** Mammal Collection | **Size:** ~1,300 voucher specimens (fluid, skin, skull) with ~950 bat specimens (and approximately 500 being catalogued) including ~20 holotypes and paratypes  **Composition:** mainly specimens of Chiroptera, ca. 73%, with ca. 25% of Rodentia, and ca. <3% of other medium and large mammals  **Strength:** The collection covers ca. 70% of known bat species in mainland SE Asia. | **Size:** ca. 2,500 tissue samples, mostly wing and liver samples  **Composition:** Preserved in ethanol tubes and stored in -80 freezer.  **Strength:** Representatives of bat fauna from mainland SE Asia, particularly Thai Peninsula and Myanmar | - Ectoparasites - Leptospira - Malaria |
| **QCAZ**  Mammal  Collection | **Size**: >18,000 specimens  **Composition:** Skin, skull, post-cranial skeletal, fluid-preserved whole organisms, frozen tissue, ectoparasites  **Strengths:** Largest collection of Ecuadorian Mammals, most species represented. | **Size**: >20,000 specimens  **Composition:** majority of the samples are frozen (-80 C), but the collection also contains samples in ethanol and lysis buffer  **Strengths:** No information provided. | - Studies are being carried out in collaboration with foreign partners (Bernhard Notch Research Institute, MSB) and with other Ecuadorian universities |
| **RMCA**  Fish  Collection | **Size**: ca. 1,000,000 specimens  **Composition:** mostly freshwater fishes from Africa  **Strengths:** Probably largest collection of African freshwater fishes; collections from nearly all parts of Africa, but mostly from Central and West Africa. Long historical record dating back to 1897. | **Size**: several ten thousands  **Composition:** mostly freshwater fishes from Africa  **Strengths:** Collections from many parts of Africa, but mostly from the Congo basin, from many poorly-explored areas. | - Monogenean ectoparasitic flatworms |
| **RMCA**  Entomology Collection | **Size**: about 300 standard insect drawers & 200 slides boxes, comprising 60-70,000 identified specimens. Additional material in ethanol.  **Composition:** The above concerns only pinned and slide preserved specimens of the Dipteran families Ceratopogonidae, Culicidae, Glossinidae, Psychodidae and Simuliidae (the entire entomological collection comprises about 6 million specimens).  **Strengths:** Strong focus on Central African material. Historical records and type material. | - No dedicated tissue collection for groups of medical or veterinary importance. | - No ongoing research on groups of medical or veterinary importance. |
| **RMCA**  Invertebrate collection | **Size**: ~13,000 gastropods on ethanol (recent and historical); >300 African adult trematode specimens on ethanol (historical collection); 3,350 records of Ixodidae in ethanol  **Composition:** Ethanol (the dry historical shell collection is not included here).  **Strengths:** trematodes & gastropods of medical importance, collected in Africa, some longitudinal collections (10 countries); ticks as potential vectors | **Size**: ~3,000 gastropod DNA extracts;  ~ 5,000 larval trematodes preserved on Whatman FTA cards, on ethanol or as DNA extracts  **Composition:** Gastropod DNA frozen or dried, but initially preserved in ethanol. Larval trematodes preserved on Whatman FTA cards, ethanol, or as DNA extracts.  **Strengths:** trematodes & gastropods of medical importance, collected in Africa, some longitudinal collections (10 countries) | - Gastropods - Trematodes |
| **RMCA**  Mammal  Collection | **Size**: ~140,000 catalogued morphological specimens (skins, skulls, skeletons, fluid-preserved material) including >720 name-bearing types.  **Composition:** Collections include specimens representing 19 (65%) of 29 recent orders, 74(48%) of 153 recent families.  **Strengths:** large series, mostly Central Africa, coordinates and localities are usually referenced | **Size**: 75,815 alcohol preserved specimens  **Composition:** with a large proportion of chiroptera, shrews (Soricidae) and rodents;  37,408 skin specimens, with a large proportion of artiodactyls, carnivores, chiroptera, primates, shrews (Soricidae) and rodents  **Strengths:** medata (specimen locality coordinates) | - No ongoing pathobiological research. |
| **TTU**  Mammal  Collection | **Size**: >150,000 voucher specimens representing >1,440 species  **Composition:** No information provided.  **Strengths:** Strong representation from the former Yugoslavia, Paraguay, Mexico, Peru, Ecuador, Honduras, Malaysia, Ukraine, and other regions of the world, as well as Texas and the southwestern U.S. | **Size:** >400,000 samples  **Composition:** The majority of the samples are frozen, but the collection also contains samples in ethanol, lysis buffer, and DNA.  **Strengths:** No information provided. | - Arenaviruses - Hantaviruses |
| **UACH**  Mammal  Collection | **Size**: > 10,000 specimens  **Composition:** skin, skull, post-cranial skeletal, fluid-preserved whole organisms, tissue samples, and karyotype test slides  **Strengths:** Strong representation of Chilean mammals. Our holdings represent the best available series of several species. | **Size**: > 10,000 samples of > 3,000 specimens  **Composition:** mostly alcohol preserved samples; some frozen samples; some RNAlater preserved samples.  **Strengths:** Holdings include the only available samples for some mammal species. | - No information provided. |
| **UMMZ**  Mammal Collection | **Size**: ~130,000 specimens  **Composition:** 29 orders; 166 families;1,366 genera; 5,532  species  **Strengths:** Representation is broad and deep, including  largest *Peromyscus* collection known. | **Size**: ~10,000 samples  **Composition:** All samples maintained in vapor-phase liquid nitrogen biorepository. Initial preservation methods vary.  **Strengths:** Samples include tissue collection from European *Mus* hybrid zone. | - Currently surveying for hantaviruses. |
